# The Interaction between Central and Peripheral Processing in Chinese Handwritten Production: Evidence from the Effect of Lexicality and Radical Complexity

**DOI:** 10.3389/fpsyg.2017.00334

**Published:** 2017-03-13

**Authors:** Qingfang Zhang, Chen Feng

**Affiliations:** ^1^Department of Psychology, Renmin University of ChinaBeijing, China; ^2^Key Laboratory of Behavioral Science, Institute of Psychology, Chinese Academy of SciencesBeijing, China

**Keywords:** handwritten production, lexicality, central processing, peripheral processing, radical boundary effect, cascadedness

## Abstract

The interaction between central and peripheral processing in written word production remains controversial. This study aims to investigate whether the effects of radical complexity and lexicality in central processing cascade into peripheral processing in Chinese written word production. The participants were asked to write characters and non-characters (lexicality) with different radical complexity (few- and many-strokes). The findings indicated that regardless of the lexicality, the writing latencies were longer for characters with higher complexity (the many-strokes condition) than for characters with lower complexity (the few-strokes condition). The participants slowed down their writing execution at the radicals' boundary strokes, which indicated a radical boundary effect in peripheral processing. Interestingly, the lexicality and the radical complexity affected the pattern of shift velocity and writing velocity during the execution of writing. Lexical processing cascades into peripheral processing but only at the beginning of Chinese characters. In contrast, the radical complexity influenced the execution of handwriting movement throughout the entire character, and the pattern of the effect interacted with the character frequency. These results suggest that the processes of the lexicality and the radical complexity function during the execution of handwritten word production, which suggests that central processing cascades over peripheral processing during Chinese characters handwriting.

## Introduction

Most research focuses on the processes and mechanisms underlying speech production, while less work has been devoted to understanding written word production. Writing involves different processing levels from the intention of writing to the actual movement execution. The current view of speech production provides a general theoretical framework from which hypotheses specific to writing can be derived (Bonin et al., 1997, 1998a,b, 2013; Rapp et al., 1997; Bonin and Fayol, 2000; Baus et al., 2013; Damian and Qu, 2013; Zhang and Wang, 2014). Recently, research in the field of written production has addressed the role of phonology codes in spelling or writing the names of pictures (Bonin et al., 2001; Afonso and Alvarez, 2011; Qu et al., 2011; Shen et al., 2013; Zhang and Wang, 2015), and these studies used writing latency as the dependent variable to uncover the planning process before writing execution. Another approach investigated the process of writing execution from a motor perspective (van Galen, 1991), and these studies utilized stroke duration or stroke velocity in writing execution as the dependent variables to explain the movement processes involved in written word production. These two approaches investigate central (or planning) and peripheral (or execution) processing, respectively. Central processing involves how we retrieve orthographic codes in our mental lexicon through the lexical or sublexical routes (Bonin et al., 2001) and their storage in working memory (Hillis and Caramazza, 1989). Peripheral processing involves the selection of allographs, the planning the sequences of letters and the execution of motor programmes (Ellis, 1982). To date, little is known about the relationship between central and peripheral processing when we write words; therefore, in the present study, we investigated the interaction between these two processes in Chinese written word production by integrating two types of measures (latency and stroke velocity).

One hypothesis assumes that that central and peripheral processing are dissociative (Baxter and Warrington, 1986; Purcell et al., 2011; Planton et al., 2013). Patients with peripheral dysgraphia have a difficulty with motor production but they can spell correctly (Baxter and Warrington, 1986). Purcell et al. (2011) carried out the first quantitative meta-analysis to distinguish central and peripheral processing using activation likelihood estimation (ALE) methods, and determined that distinct neural networks are involved in central and peripheral processes of written word production (see also Planton et al., 2013 for a similar conclusion).

The opposite hypothesis assumes that central and peripheral processing are cascaded. Two different approaches have been employed to investigate the interaction among the processes involved in written word production. One approach examines whether peripheral processing is affected by factors that regulate central processing (Zesiger et al., 1993; Delattre et al., 2006; Kandel et al., 2013; Roux et al., 2013). Zesiger et al. (1993) found that there is a facilitation effect of words over pseudowords and of frequent trigrams over nonfrequent trigram, and pseudowords ending with a nonfrequent trigram in adults, in central processing. However, lexicality and trigram frequency do not affect the writing duration and the trajectory length of writing in children, in peripheral processing. Delattre et al. (2006) examined the effects of word frequency and orthographic regularity in a spelling to dictation task. Results revealed that both factors affect writing latencies, which reflects central processing. For the peripheral processing, writing irregular words yielded longer writing durations compared to writing regular words, but word frequency did not yield a significant effect. The authors concluded that orthographic regularity regulate central and peripheral processing of handwriting, and suggested that spelling processes cascade onto motor processes. Kandel et al. (2013) manipulated germination within a word pairs to examine the influence of the orthographic properties on the handwriting movement. The word pairs shared the initial letters and differed on the presence of a doublet at the same position (i.e., DISSIPATE vs. DISGRACE). Results revealed that latencies were shorter for words with double letters than control words. Importantly, the impact of double letters was also observed during writing execution, with shorter letter writing durations and intervals for words with double letters than controls. Therefore, double letters affect central processing as well as peripheral processing. Roux et al. (2013) examined the interaction between central and peripheral processes of writing in French participants who were asked to write regular words, irregular words and pseudo-words. The authors observed that pseudo-words resulted in longer writing latencies than regular words and that letter durations were longer for irregular words than for regular words. These findings indicate that movement duration can be affected by factors that regulate central processing. Therefore, Roux et al. suggested that central processing of the conflict generated by lexically specific and assembled spelling information for irregular words is not entirely resolved when peripheral processing begins. Furthermore, these results give rise to the possibility that higher order linguistic variables can affect peripheral processing (Bonin et al., 2013; Roux et al., 2013), which supports the view that activation cascades from central processing to peripheral processing in written word production.

Another approach investigates the relationship between semantics and orthography at the preparation stage of written word production (Roux and Bonin, 2011; Qu and Damian, 2015). Roux and Bonin (2011) investigated how information flows within the lexical system of central writing processing using a picture-picture priming paradigm in French. Participants named target pictures that were accompanied by context pictures that phonologically and orthographically related or unrelated names. The authors observed that the writing latency was shorter with a distractor picture that was orthographically related (shared the initial letter but not the initial sound) to the target compared to the unrelated condition, while a distractor picture that was phonologically related (shared the initial phoneme but not the initial grapheme) to the target did not produce a reliable effect. Additionally, an object identification task and a semantic categorization task were used, and the difference between the related and unrelated distractor conditions was not significant in both tasks. These findings ruled out a perceptual and conceptual account of the orthographic effect. The authors suggested that activation within the lexical system involves a cascaded pattern in writing.

Qu and Damian (2015) addressed the same issue in Chinese written word production. They employed a Stroop task in which the Chinese participants were presented with colored objects and instructed to write down the name of the color while ignoring the object. In the experiment, the critical manipulation was the orthographically related condition. Each object was combined with a color such that the color name (i.e., 橙, /cheng2/, orange as the target name) shared a radical with the first character of the object name (i.e., 枕头, /zhen3tou2/, pillow as the non-target word). They found a significant facilitation effect in the orthographically related condition, in comparison with the orthographically unrelated condition. This finding indicates that the non-target lexical nodes activate their corresponding orthographic representation and that activation flows in a cascaded fashion in Chinese written word production.

## The present study

We used the first approach to investigate the interaction between central processing and peripheral processing in Chinese written word production. The interaction between central and peripheral processing has not been investigated in Chinese by this approach in adults, which is important given the differences in the orthographic representation between alphabetic and non-alphabetic scripts (i.e., Chinese).

### The syllable boundary effect in non-alphabetic scripts

The syllable boundary effect in handwriting production has been confirmed across studies, and syllables are taken as one of the processing units in central and peripheral processing. In a copying task, Kandel and Valdois (2006a) observed that, for French 1st to 5th graders, the gesture of the first syllable has been programmed before movement execution. The child yielded a movement time peak at the syllable boundary (name it syllable boundary effect), indicating that they programmed the second syllable on-line. Kandel and Valdois (2006a) thus suggested that the primary students employ syllables as units in central and peripheral processing. The syllable boundary effect in handwriting production has been replicated in other developmental studies in Children (Kandel et al., 2006b; Kandel and Valdois, 2006b) and in adults (Kandel et al., 2006a; Lambert et al., 2008).

Kandel et al. (2009) further examined whether the syllable the children use to segment words is determined phonologically or orthographically. Third, 4th, and 5th graders were asked to write words that were mono-syllables phonologically but bi-syllables orthographically, and these words were matched to words that were bi-syllables both phonologically and orthographically. They observed that the children yielded significant peaks at the syllable boundary on letter stroke duration and fluency, reflecting that the children use orthographic rather than phonological syllables as processing units to plan writing including central and peripheral processes.

Studies revealed that the syllables boundary effect is interactive with other linguistic variables (i.e., lexicality) in written production (Transler et al., 1999; Kandel and Valdois, 2006a). Transler et al. (1999) asked participants (normal and hearing impaired children) to copy words and pseudo-words, and the properties of syllable boundary position, number of syllables and orthographic similarity were varied. They found that real- and pseudo-words had different syllable boundaries in the orthographically similar words. Kandel and Valdois (2006a) used a copying task to study spelling acquisition, and children were asked to copy words and pseudo-words. They observed that first and second graders' children yielded fewer gaze lifts (recorded by a camera) in words than pseudo-words. This finding indicated that the lexicality influences the processing of written production. However, little is known how the lexicality affects written production in Chinese.

### The radical boundary effect in chinese

Chinese characters are composed of strokes that contain two-dimensional spatial information, which can be divided at different levels: strokes, logographemes and radicals (see UCS Chinese character database; Standards Press of China, 1994; Shu, 2003). For example, the character “枝” (branch, /zhi1/) can be divided into two parts: the semantic radical “木” and the phonetic radical “支.” The semantic radical provides clues to the meaning of the character, while the phonetic radical provides clues to the pronunciation of the character. Here, the phonetic radical “支” can be further divided into two logographemes: “十” and “又.” Studies have shown that the semantic and the phonetic radicals affect word comprehension processing (Law, 2004; Law et al., 2005; Bi et al., 2007).

For orthographic representation, radicals are processing units in Chinese word recognition (Feldman and Siok, 1999; Ding et al., 2004; Hsiao et al., 2006, 2007; Tsang and Chen, 2009; Su et al., 2012). For example, in Tsang and Chen's (2009) study, for each trial, participants were first shown two Chinese characters as primes [i.e., “秋” (qiu1, autumn in English) and “吐” (tu4, spit)], and then were shown a target character “和” (he2, and) that shared “禾” with “秋” and “口” with “吐” in the related condition; whereas the target characters in the control condition did not share any radicals with the two prime characters. Participants were asked to decide whether a target character was the one of two primes. The results showed that participants made more errors in the related condition compared to the control condition. Feldman and Siok (1999) found that the latencies in the character decision task were longer when the target and its prime shared one radical in comparison to when the target and its prime did not share any radicals. Ding et al. (2004) investigated the mental representation of radicals using a priming task in Chinese. In each trial, participants were presented prime (in which the prime was a character that was a constituent radical of the target character or unrelated prime), followed by the target character, they were asked to determine whether the target was a real Chinese character. The authors found that there is a facilitation effect when the primes shared a radical with the targets in the same position (see also Tsang and Chen, 2009). These findings show that radicals function as units of processing and affect the recognition of Chinese characters.

Radicals have also been shown to function as a unit in Chinese written word production. Han et al. (2007) found that in dysgraphia, more errors involve radical substitution or deletion. Similar findings were observed in 2nd grade students (mean age: 7.5 years) in primary school (Shi et al., 2011). Using chronometric measures in an implicit priming paradigm, Chen and Cherng (2013) examined written word production processing in adults. First, the participants learned prompt-target associative word pairs, and then wrote down the corresponding target word when they were presented with a prompt word. The overlap conditions among the target words included the first stroke shared, the first and the second strokes shared, the first logographemes shared, the first radical shared or no shared conditions. The authors found that the conditions of shared logographemes and shared radicals significantly facilitated writing latency, while the condition of shared strokes had no effect.

In the present study, we measured the writing latency and the velocity of each stroke to investigate the pattern of activation spreading between central and peripheral processing in Chinese handwriting. As mentioned above, radicals are the processing unit in word recognition (Feldman and Siok, 1999; Hsiao et al., 2006, 2007; Tsang and Chen, 2009; Su et al., 2012) and in written word production (Han et al., 2007; Shi et al., 2011; Chen and Cherng, 2013). The number of strokes in Chinese characters affects word reading (Giovanni, 1994; Ding et al., 2004). For orthographic processing in written production, it is necessary to chunk strokes into radicals for outputting orthographic codes smoothly. Kandel and Valdois (2006a) observed that the children programmed the second syllable on-line. To examine whether or not the 2nd radical is programmed before writing execution, we therefore manipulated the number of strokes in the study as well. If participants encoded the 2nd radical before writing execution, we expect the number of strokes yield influence on writing latencies, characters with the many-strokes would yield longer latencies than those with the few-strokes.

**Figure 1:**
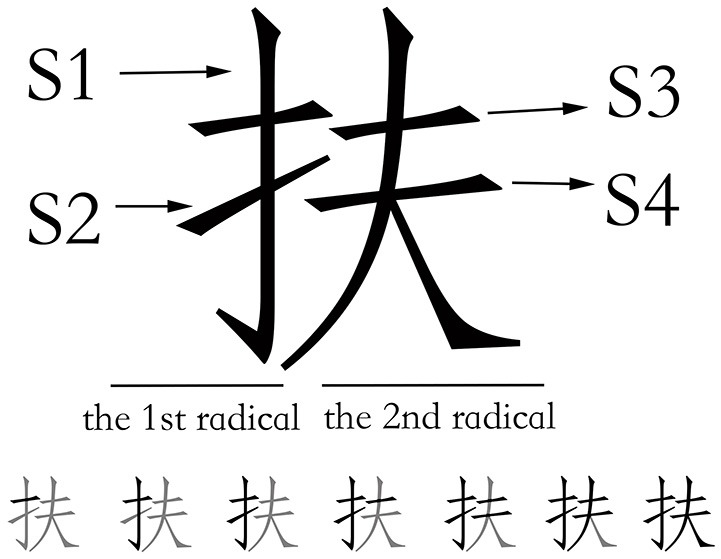
**An example of the strokes in the within and between radicals and the strokes order during writing (The black indicates the strokes are written, and the gray indicates the strokes are not written)**.

To examine the radical boundary effect in peripheral processing, we selected four strokes at different positions within a character (see Figure 1 for an example). Stroke 1 (S1) and S2 were the last two strokes in the first radical, and S3 and S4 were the first two strokes in the second radical, which means that, for writing sequence, S1 and S4 were strokes not at the radical's boundary (non-radical boundary strokes), while S2 and S3 were strokes at the radical's boundary (radical boundary strokes). Given that the strokes in Chinese characters have different lengths and that the size of the participants writing output were different, we did not considered writing duration as a measure of writing movement; instead, we measured the average velocity of each stroke. By comparing the velocity of four strokes, we determined whether there was a radical boundary effect during the writing execution.

The aim of the present study was to investigate how the complexity of the radical (few- and many-strokes), the stroke position (non-radical boundary and radical boundary strokes) and the lexicality (experiment 2) affect both central and peripheral processing in writing. For central processing, we expect that the latency would be longer in the many-strokes condition compared to the few-strokes condition. For peripheral processing, we expected that we would observe a radical boundary effect in Chinese that is similar to the syllable boundary effect in alphabetic languages (Kandel and Valdois, 2006a; Kandel et al., 2006a). Furthermore, we predict that because the processing load would increase at the radical boundary strokes, the velocity of the strokes would be slower for S2 and S3 compared to S1 and S4.

## Ethics statement

The current study was approved by the Independent Ethics Committee of the Institute of Psychology, Chinese Academy of Sciences in Beijing. Written consent was obtained from participants before the administration of the experiments.

## Experiment 1

### Methods

#### Participants

A total of 20 students (11 males with an average age of 23.7 years and ranging from 20 to 27 years) were recruited from Beijing Forest University and China Agricultural University and were paid approximately $4 to participate in the study. They were all native Chinese speakers, with normal or corrected-to-normal vision.

#### Materials

For this study, 30 Chinese characters were selected. Each Chinese character had a left-right structure and consisted of two radicals. For example, “抹,” which means wipe, has a left radical “扌” and a right radical “末.” We manipulated the stroke number of the second radical in each Chinese character, and there was significant difference between the few-strokes and the many-strokes characters (*t* = 7.23, *p* < 0.001) (see Table 1 for the mean of the 2nd radicals). The two sets of characters were matched based on the character frequencies, syllable frequencies, regularity, homophone numbers and the stroke number of the first radical (see Table 1). The regularity of a Chinese character indicates whether the phonetic radical can provide a clue to the whole character's pronunciation. There were 7 irregular characters and 8 regular characters in the few-strokes condition, and 8 irregular characters and 7 regular characters in the many-strokes condition. Statistical analysis showed that there were no differences between the two groups (few-strokes condition and many-strokes condition) in the character frequency, syllable frequency and homophone number (all *ts* ≤ 0.6, *ps* ≥ 0.5).

**Table 1:** **Properties of the different stimuli used in experiments 1 and 2**.

**Properties**	**Radical complexity**			
	**Experiment 1**		**Experiment 2**	
	**Few**	**Many**	**Few**	**Many**
Syllable frequency	971.6	1,264.6	682.9	782.2
Homophone number	27.2	25.8	–	–
Character frequency	12.5	11.7	–	–
Strokes of 1st radicals	3.2	3.1	2.8	6.4
Strokes of 2nd radicals	3.5	5.8	2.6	6.8
Number of regular characters	8	7	3	5
Number of irregular characters	7	8	13	11

#### Design

The experimental design included radical complexity of the second radical (few- vs. many-strokes) and stroke positions (S1, S2, S3, and S4) as the within-participants factors. Within an experimental block, each participant was asked to write down the 30 target characters. This block was repeated two times; therefore, the entire experiment consisted of 60 trials. The sequence of target characters in each block was randomized.

#### Apparatus

The experiment was programmed and executed using the handwriting software, Ductus (Guinet and Kandel, 2010). The participants wrote down the characters using a specialized pen (Inking pen) on lined paper attached to the digitizer (Wacom Intuos 4; sampling frequency: 200 Hz, accuracy: 0.02 mm), which was connected to a computer that monitored the executed movements of the participants.

#### Procedure

The participants were individually tested in a quiet room at a comfortable viewing distance from the computer. Before the experiment started, the participants were instructed that their task was to write the characters.

During the experiment, each trial began with the presentation of a fixation point (+) at the middle bottom of the screen for 500 ms, which was followed by a blank screen for 500 ms. Next, a Chinese character (24 point font) was presented and remained on the screen until the subjects began to write on the tablet. The next trial began after the experimenter saw that the participant had completed the response and pressed a number key. All of the characters were presented in random order across the participants.

The characters were displayed at the bottom of the screen to reduce the head and eye movements of the participants as they wrote. During the experiment, the participants were asked to write in a normal speed while paying attention to lifting the pen between each stroke so that we could clearly determine the beginning and end of each stroke. The participants were instructed to hover the stylus just above the corresponding line on the sheet in anticipation of the response to prevent unnecessary arm movement during each response. Furthermore, the participants were asked to initiate writing the characters as accurately and quickly as possible. The experiment consisted of 2 blocks, and each block consisted of four warm-up trials and 30 target characters with a break between each block. During the experiment, participants could not see their writing trajectory on the computer screen to avoid the influence of visual feedback.

#### Data analysis

Ductus is a semi-automatic handwriting analysis software (see Guinet and Kandel, 2010 for information on the analysis procedure). The data were smoothed using a finite impulse response filter (Rabiner and Gold, 1975) with a 12 Hz cut-off frequency. We measured the writing latencies between the presentation of target item and the onset of handwritten production. The Chinese characters were segmented into strokes according to the fixed sequence as illustrated in Figure 1. The stroke duration was measured as the time the participants took to write each stroke. The stroke length was measured as the distance between the beginning and the end point of each stroke for each participant. The velocity of each stroke was calculated using the following equation:

Stroke velocity = stroke length / duration

When the cognitive system is overloaded, movement slows (i.e., dysfluence), which results in an increase in the stroke duration. Therefore, the duration for each stroke should increase at specific positions (i.e., at the radicals boundary strokes).

### Results

We used the lmer program of the lme4 package to estimate the fixed effects and parameters of the LMM (Linear Mixed Model) (Bates, 2005; Baayen et al., 2008) using R software (R Development Core Team, 2009). The data were conformed to the Gaussian distribution. The data were analyzed using a linear mixed-effects model (*lmertest* package) that included the fixed effects of radical complexity (few- and many-strokes) and the by-participant and by-item random intercepts. The models were fit to the data using restricted maximum likelihood estimation, which seeks to find the parameter values that make the model's predicted values most similar to the observed values. Model fitting was performed by initially specifying a model that included only the random factors (participants and items) and was then enriched by subsequently adding the fixed factors of radical complexity. The best-fit model was defined to be the most complex model that significantly improved the fit over the previous model. A fixed factor was determined to have no significant influence on the dependent variables (i.e., writing latencies and stroke velocity) when adding a fixed factor did not significantly improve the fit. The function of *summary()* was used to obtain *p*-values of significance.

#### Writing latencies

Data from the incorrect responses (including the wrong characters, the wrong strokes and characters containing 2 or more strokes produced in one trajectory; 7.4%), writing latencies longer than 1,500 ms or shorter than 300 ms (1.8%), and latencies more than 2.5 standard deviations from the mean (2.2%) were removed from the analyses.

The best-fit model did not include any variables. Adding radical complexity did not significantly improve the fit [$\chi_{\left(1,\;1150\right)}^{2}$ = 3.14, *p* = 0.08]. The average latency in the few-strokes condition (653 ms, *SD* = 190 ms) was longer than in the many-strokes condition (641 ms, *SD* = 188 ms); however, the difference was not statistically significant (*t* = 1.8, *p* = 0.08), which suggests that the latencies were comparable between the few- and many-strokes conditions.

In order to determine the contributions of the first radicals and the second radicals, we calculated the correlation between the writing latency and the number of strokes in the first radical, and the correlation between the writing latency and the total number of strokes of a whole character. Results indicated that both correlations were not significant (both *rs* < 0.22, *ps* > 0.25), reflecting that the number of strokes did not yield significant influence on the writing latency.

#### The stroke velocity of the writing execution

The present analysis aims to examine the radical boundary effect in writing movement. As previously described, we selected four strokes at different positions within a character (see Figure 1 for an example). Therefore, an additional independent variable (the stroke position) was included in the analysis of the stroke velocity. Model fitting was performed by initially specifying a model that included only the random factors (participants and items) and was then enriched by subsequently adding the fixed factors of the radical complexity, the stroke position, and the interaction between the radical complexity and the stroke position one by one. For the stroke velocity, the best-fit model included the stroke position and the interaction between the radical complexity and the stroke position. Adding the radical complexity did not improve the fit [$\chi_{\left(1,\;3876\right)}^{2}$ = 1.95, *p* = 0.16]. The model shows that the effect of the stroke position is statistically significant (S1–S2: *t* = −8.12, *p* < 0.001; S1–S3: *t* = −7.01, *p* < 0.001; S1–S4: *t* = 6.50, *p* < 0.001), as well as the interaction effect between the radical complexity and the stroke position (*t* = 3.54, *p* < 0.001). Table 2 presents the LMM estimates of the fixed effects for stroke velocity of writing execution.

**Table 2:** **The LMM estimates of the fixed effects for stroke velocity of the writing execution in experiment 1**.

**Fixed effects**	**Estimates**	**SE**	***t*-value**
(Intercept)	8.31	0.52	26.55
S2	−1.22	0.15	−8.12^***^
S3	−1.05	0.15	−7.01^***^
S4	0.98	0.15	6.50^***^
S2:the many-strokes	0.07	0.22	0.32
S3:the many-strokes	0.76	0.22	3.54^***^
S4:the many-strokes	−0.28	0.22	−1.28

*** *p < 0.001*.

**Figure 2:**
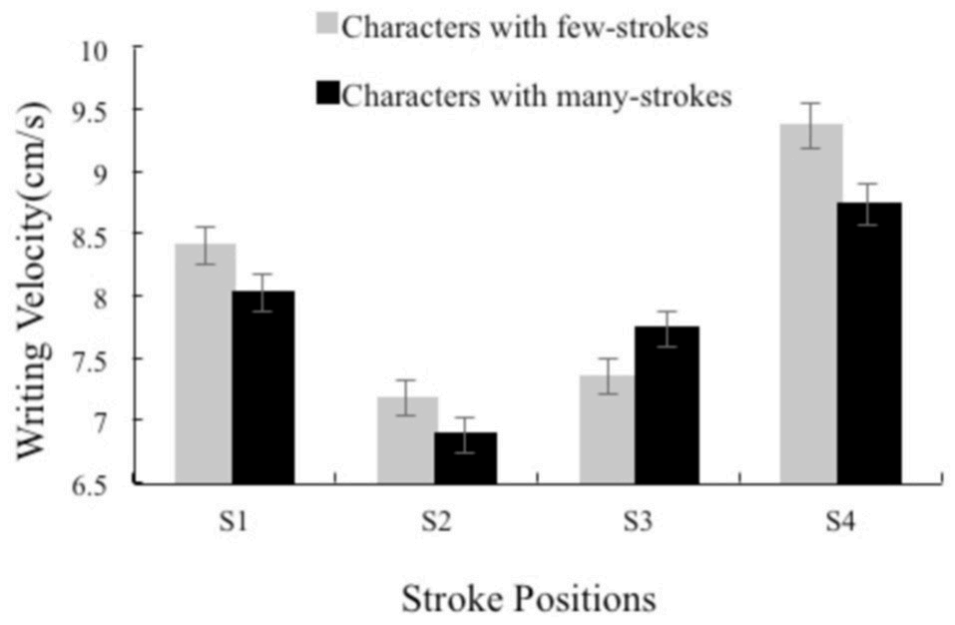
**The average writing velocity (mm/s) as a function of the stroke position (S1, S2, S3, and S4) and the radical complexity in experiment 1**.

We conducted multiple comparisons among the stroke positions (S1, S2, S3, and S4) using the Bonferroni correction method in the few- and many-strokes conditions, respectively. We compared the strokes at the radical boundary (S2 and S3) and the strokes not at the radical boundary (S1 and S4), respectively. We also compared the S2 and S3 across two radicals (S2 vs. S3). A total of 5 *t*-tests were performed in each condition; therefore, a *p*-value of less than 0.01 is considered significant for this analysis. For the few-strokes condition, the results indicated that the stroke velocity of S2 and S3 was significantly slower than the stroke velocity of S1 (S2–S1: *t* = −8.76, *p* < 0.001; S3–S1: *t* = −7.10, *p* < 0.001). The velocity of S3 was as slow as the velocity of S2 (*t* = 1.18, *p* = 0.24). The velocity of S4 was significantly faster than the velocity of both S2 and S3 (S4–S2: *t* = 14.03, *p* < 0.001; S4–S3: *t* = 16.71, *p* < 0.001). Finally, the velocity of S2 and S3 were the slowest among the 4 strokes. For the many-strokes condition, the results indicated that the velocity of S2 was significantly slower than the velocity of S1 (S2–S1: *t* = −8.75, *p* < 0.001). The velocity of S3 was not significantly slower than the velocity of S1 (S3–S1: *t* = −2.05, *p* = 0.05). The velocity of S3 was significantly faster than the velocity of S2 (*t* = 6.34, *p* < 0.001). The velocity of S4 was significantly faster than the velocity of both S2 and S3 (S4–S2: *t* = 12.69, *p* < 0.001; S4–S3: *t* = 7.84, *p* < 0.001). Figure 2 shows the average velocity as a function of the stroke position (S1, S2, S3, and S4) and the radical complexity in experiment 1.

### Discussion

We found a significant velocity decrease for the radical boundary strokes, which indicated that there was an increase of processing load at the radical boundary strokes during writing execution. Because the radical is an output unit during writing movement, the participants prepared to process the second unit. This processing lasts from the last stroke of the first radical to the first stroke of the second radical. Our findings are in agreement with the findings by Kandel and Valdois (2006a), which observed that the writing duration is longer for the last letter of the first syllable compared to the other letters in French. This result suggests that anticipatory processing occurs during handwriting movement. The participants processed the information from the next functional unit while they were writing the current unit. This cascaded pattern in written word production has been consistently observed in studies (Magnussen et al., 1996; Roux and Bonin, 2011; Bertram et al., 2015).

It is noteworthy that the velocity was slower at S3 than at S1 or S4, indicating that the cognitive system was more demanded at the radical boundary strokes than at the non-radical boundary strokes. We thus suggest that the retrieval of the information from the second radical begins after the last stroke of the first radical and persists until the first stroke of the second radical. This finding is in contrast to Kandel and Valdois (2006a), who only observed the syllable boundary effect at the last letter within a syllable, which might indicate that a different boundary effect occurs in Chinese writing movement because a typical Chinese character involves complex stroke combinations and complicated spatial structure.

For the simple characters S2 was as slow as S3, which might reflect that the participants process the first and the second radicals equivalently. Whereas for the complex characters, S3 was significantly faster than S2 might reflect that the participants process the first and the second radicals differently. The simple characters consist of the equal small number of strokes in the first and the second radicals (both are less than 4), whereas the complex characters consist of less strokes in the first radicals (less than 4) but more strokes in the second radicals (more than 5 and less than 8). Due to the complexity of the second radical in the complex characters, the patterns of writing execution were distinct between simple and complex characters. This finding suggests that the participants' writing execution was dynamic which depends on the properties of radicals and strokes within a character.

The writing latencies were equivalent in the few- and many- strokes conditions. One possibility is that the difference between the two conditions was too minor to approach significance. Another possibility is that the participants only prepared the first radical before the initiation of writing movement. Due to similar strokes in the first radical in both conditions, similar writing latencies were observed.

## Experiment 2

As mentioned in the introduction, compared to the real words, the lexicality affects the syllable boundary effect for the pseudo-words in alphabetic languages such as French. Therefore, in experiment 2, we aimed to examine the effects of the radical complexity, the lexicality and their interaction on writing preparation and movement in Chinese written word production.

### Methods

#### Participants

Twenty-three students (12 males with an average age of 22.5 years and ranging from 19 to 25 years) were recruited from Beijing Forest University and China Agricultural University and were paid approximately $4 to participate in the study. They were all native Chinese speakers, with normal or corrected-to-normal vision.

#### Material

For this study, 32 Chinese characters were selected. Each Chinese character had a left-right structure and consisted of two radicals. The characters were divided into two groups according to the number of strokes in the character: few- and many-strokes conditions. To exclude the effect of character familiarity, we selected real rare characters, and all the participants reported that they had not see the characters before. The two sets of characters were matched based on syllable frequency, the number of regular and irregular characters within a group (see Table 1 right panel). The non-characters were composed by transposing the radicals used in the real characters. The radicals (i.e., 亻, 氵, 犭, 𧾷, 舟) always appear in the left position within a character, whereas the radicals (i.e., 里, 良) almost always appear in the right position. The real characters bear this type of radical structure, whereas the non-characters didn't have legal radical structure. In other words, the radicals' positions were legal in the real characters but illegal in the non-characters. For example, “屸” and “扖” were two real characters, and the “山” in “屸” was combined with the “入” in “扖” to create the non-character (see Appendix in Supplementary Material for details). The radicals in the few-strokes and the many-strokes condition were re-combined to create the non-characters in the corresponding conditions. Therefore, the number of strokes in the characters and non-characters were identical.

#### Design

The experimental design included radical complexity (few- vs. many-strokes), lexicality (real vs. non-characters) and stroke position (S1, S2, S3, S4) as the within-participants factors. In one block, the participants were asked to write 32 target characters with 4 characters as a warm-up trial, whereas in the second block, the participants wrote the 32 target non-characters with 4 non-characters as a warm-up. The sequence of the target items in each block was randomized. The participants were required to write each character or non-character three successive times.

#### Apparatus and procedure

The apparatus and procedure were identical to experiment 1.

#### Data analysis

Experiment 2 used a paradigm different from that used in experiment 1 (Lambert et al., 2008). Participants were asked to copy Chinese characters or non-characters that appear on the computer screen. They wrote each item three times on a paper, which was attached to a digitizer tablet. We measured the writing latencies between the presentation of target item and the onset of the first handwritten production (latency 1), the time interval between the offset of the first production and the onset of the second production (latency 2), and the offset of the second production and the onset of the third production (latency 3). Compared to the first latencies, the second and third latencies did not involve visual encoding of the target items, but mainly focused on spelling activation and movement programming (Lambert et al., 2008). Because the movement length for the interval among the three repetitions were different, we calculated the velocity as the participants shifted the pen from the offset of the first production to the onset of the second production (the shift velocity from 1 to 2), and the velocity as the participants shifted the pen from the offset of the second production to the onset of the third production (the shift velocity from 2 to 3). The movement length was obtained using the Ductus software, which can record the position information of beginning and ending points. The shift velocity among three repetitions was calculated using the following equation:

Non-stroke shift velocity = non-stroke movement length/time interval.

The first latencies, the shift velocity from 1 to 2, and the shift velocity from 2 to 3 were indicative of central processing. We investigated writing processing without the interference of visual recognition by analysing the shift velocities among the three repetitions.

Similar to experiment 1, the velocity of the different stroke positions (S1, S2, S3, and S4) were also measured, which were indicative of peripheral processing during written word production.

### Results

Similar to experiment 1, the model fit was performed by initially specifying a model that included only the random factors (participants and items) and was then enriched by subsequently adding the fixed factors of lexicality, radical complexity, and stroke position one by one, which was followed by the interaction between two factors and then the 3-way interaction among the three fixed variables. The best-fit model was defined to be the most complex model that significantly improved the fit over the previous model. A fixed factor was determined to have no significant influence on the dependent variables (i.e., naming latency, shift velocity or writing velocity) when adding a fixed factor did not significantly improve the fit.

#### The writing latencies and shift velocity among the repetitions

The data from the incorrect responses (3.9%) were excluded. Latencies longer than 3,000 ms or shorter than 800 ms (3.9%) and latencies that exceeded 2.5 standard deviations (1.6%) were also excluded. Because the mean latencies were 900 ms longer in experiment 2 (approximately 1,560 ms) than in experiment 1 (652 ms), and because the range of writing latencies was 594 to 8,297 ms, we included additional exclusion criteria for extreme data that was shorter than 800 ms and longer than 3,000 ms. Table 3 shows the mean writing latencies (in ms) (SD), and the average non-stroke shift velocity from 1 to 2 and from to 2 to 3 by the lexicality and radical complexity.

**Table 3:** **The mean writing latencies (in ms) and the standard deviation (SD), and the mean non-stroke shift velocity (mm/s) from 1 to 2 and from 2 to 3 by the lexicality and the radical complexity in experiment 2**.

**Measures**	**Lexicality**	**Radical complexity**	
		**Few-strokes**	**Many-strokes**
Latencies	Characters	1,429 (359)	1,692 (447)
	Non-characters	1,422 (363)	1,699 (470)
Non-stroke shift velocity from 1 to 2	Characters	10.67 (2.82)	9.81 (2.65)
	Non-characters	10.11 (2.67)	9.29 (2.73)
Non-stroke shift velocity from 2 to 3	Characters	11.36 (2.96)	10.02 (2.54)
	Non-characters	9.93 (2.64)	9.12 (3.02)

For the writing latency, the best-fit model included the radical complexity. Adding the lexicality [$\chi_{\left(1,\;1330\right)}^{2}$ = 0.30, *p* = 0.58] or the interaction between the radical complexity and the lexicality [$\chi_{\left(1,\;1330\right)}^{2}$ = 0.24, *p* = 0.62] did not improve the fit. Regardless of the lexicality, the latencies in the many-strokes condition were significantly longer compared to the few-strokes condition (*t* = 11.01, *p* < 0.001). Furthermore, the latencies were equivalent between the characters and the non-characters (*t* = 0.01, *p* = 0.99). The correlation between the writing latency and the number of strokes in the first radical was 0.800, *p* < 0.001, and the correlation between the writing latency and the total number of strokes for whole characters was 0.798, *p* < 0.001. These findings reflect that the properties of whole characters may influence the writing latency.

For the non-stroke shift velocity from 1 to 2, the best-fit model included the lexicality and the radical complexity. Adding the interaction between the radical complexity and the lexicality did not improve the fit [$\chi_{\left(1,\;1160\right)}^{2}$ = 0.06, *p* = 0.81]. The difference between the characters and the non-characters was marginally significant (*t* = 1.76, *p* = 0.08), whereas the difference between the few-strokes condition and the many-strokes condition was significant (*t* = −3.11, *p* < 0.01).

For the non-stroke shift velocity from 2 to 3, the best-fit model included the lexicality and the radical complexity. Adding the interaction between the radical complexity and the lexicality did not improve the fit [$\chi_{\left(1,\;1193\right)}^{2}$ = 0.70, *p* = 0.40]. Similar to the shift from 1 to 2, the difference between the characters and non-characters (*t* = 4.13, *p* < 0.001) and the difference between the few-strokes condition and the many-strokes condition (*t* = −4.00, *p* < 0.001) was significant.

#### The velocity of the strokes during writing execution

The best-fit model included the lexicality, the radical complexity, the stroke positions, the interaction between the lexicality and the stroke positions, the interaction between the radical complexity and the stroke positions, and the 3-way interaction among the three variables (lexicality, radical complexity and stroke position). Adding the lexicality did not significantly improve the fit [$\chi_{\left(1,\;11364\right)}^{2}$ = 2.66, *p* = 0.10]. Adding the interaction between the lexicality and the radical complexity did not significantly improve the fit [$\chi_{\left(1,\;11364\right)}^{2}$ = 0.68, *p* = 0.41]. Table 4 presents the LMM estimates of the fixed effects for stroke velocity of the writing execution in experiment 2.

**Table 4:** **The LMM estimates of the fixed effects for stroke velocity of the writing execution in experiment 2**.

**Fixed effects**	**Estimates**	**SE**	***t*-value**
(Intercept)	6.82	0.26	26.24
Character	−0.33	0.26	−1.26
Many-strokes	0.67	0.27	2.46^*^
S2	0.98	0.09	10.43^***^
S3	−0.54	0.09	−5.76^***^
S4	0.57	0.09	6.10^***^
Character:Many-strokes	−1.37	0.38	−3.57^***^
Character:S2	−0.77	0.13	−5.90^***^
Character:S3	0.56	0.13	4.30^***^
Character:S4	0.83	0.13	6.32^***^
Many-strokes:S2	−1.86	0.15	−12.22^***^
Many-strokes:S3	−1.06	0.15	−6.97^***^
Many-strokes:S4	−1.81	0.15	−11.89^***^
Character:Many-strokes:S2	2.39	0.22	10.96^***^
Character:Many-strokes:S3	0.95	0.22	4.34^***^
Character:Many-strokes:S4	0.98	0.22	4.51^***^

*** *p < 0.001*,

* *p < 0.05*.

**Figure 3:**
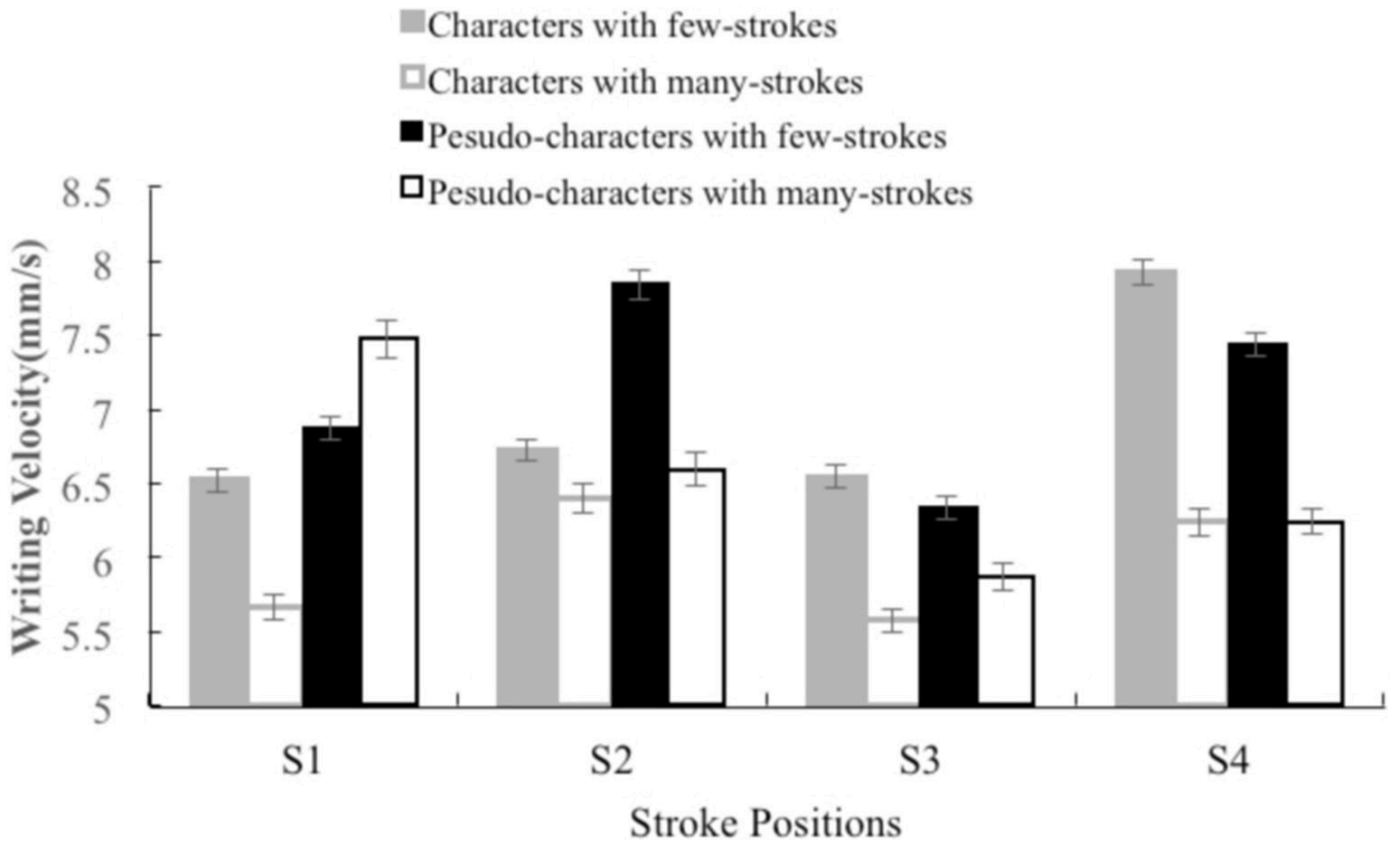
**The average writing velocity (mm/s) as a function of the stroke position (S1, S2, S3, and S4) and the radical complexity in experiment 2**.

Subsequent Bonferroni multiple comparisons on velocity of each stroke were carried out for the lexicality and the radical complexity (correction significant *p* < 0.01). Table 5 presents the *t*-values for Bonferroni multiple comparisons in each condition. We compared the strokes at the radical boundary (S2 and S3) and the strokes not at the radicals boundary (S1 and S4), respectively. We also compared the S2 and S3 across two radicals, and results indicated that S3 had the slowest velocity. For the few strokes characters, there were significant differences between strokes not at the radical boundary and strokes at the radical boundary, S1–S2: *t* = −2.41, *p* < 0.01; S2–S4: *t* = −13.5, *p* < 0.001; S3–S4: *t* = −16.11, *p* < 0.001, and significant difference between strokes at the radical boundary, S2–S3: *t* = 2.42, *p* < 0.01. For the many strokes characters, there were significant differences between strokes not at the radical boundary and strokes at the radical boundary, S1–S2: *t* = −8.17, *p* < 0.001; S3–S4: *t* = −7.34, *p* < 0.001, and significant difference between strokes at the radical boundary, S2–S3: *t* = 8.53, *p* < 0.001. For the few strokes non-characters, there were significant differences between strokes not at the radical boundary and strokes at the radical boundary, S1–S2: *t* = −10.77, *p* < 0.001; S1–S3: *t* = 6.96, *p* < 0.001; S2–S4: *t* = 4.05, *p* < 0.001; S3–S4: *t* = −13.47, *p* < 0.001, and significant difference between strokes at the radical boundary, S2–S3: *t* = 15.09, *p* < 0.001. For the many strokes non-characters, there were significant differences between strokes not at the radical boundary and strokes at the radical boundary, S1–S2: *t* = 6.86, *p* < 0.001; S1–S3: *t* = 13.57, *p* < 0.001; S2–S4: *t* = 3.23, *p* < 0.001; S3–S4: *t* = −3.83, *p* < 0.001, and significant difference between strokes at the radical boundary, S2–S3: *t* = 6.25, *p* < 0.001. Figure 3 presents the mean writing velocity (mm/s) as a function of the stroke position (S1, S2, S3, and S4) and the radical complexity in experiment 2.

**Table 5:** **The *t*-values for *Bonferroni* mulitiple comparisons on velocity of each stroke by the lexicality and the radical complexity in experiment 2**.

**Comparisons**	**Conditions**			
	**Characters**		**Non-characters**	
	**Few-strokes**	**Many-strokes**	**Few-strokes**	**Many-strokes**
Strokes not at the radical boundary vs. strokes at the radical boundary				
S1–S2	−2.41^**^	−8.17^***^	−10.77^***^	+6.86^***^
S1–S3	−0.27	+0.97	+6.96^***^	+13.57^***^
S2–S4	−13.5^**^	+1.58	+4.05^***^	+3.23^***^
S3–S4	−16.11^***^	−7.34^***^	−13.47^***^	−3.83^***^
Strokes at the radical boundary				
S2–S3	+2.42^**^	+8.53^***^	+15.09^***^	+6.25^***^

*** *p < 0.001*,

** *p < 0.01*.

*“−” indicates that the velocity of the former was slower than the latter in a comparison, “+” indicates a reverse pattern*.

## Discussion

For central processing, we found that the writing latencies was affected by the complexity of the radical, and the non-stroke shift velocity from 1 to 2 and from 2 to 3 were both affected by the lexicality and the radical complexity. Increasing the differences between the few- and the many-strokes conditions resulted in a significant difference in both the latencies and the writing velocities. The correlation analysis between the writing latency and the number of strokes indicated that the participants might prepare the whole character, not just the first radical, before they could initiate writing execution.

For peripheral processing, the writing velocity of the few-strokes characters was statistically significantly faster at S2, S3, and S4 than that of the many stroke characters. Furthermore, the writing velocity of the real characters had a different pattern from the non-characters. The velocity of S1 was as slow as the S3 in real characters, whereas the former was faster than the latter in non-characters. These findings indicate that both the lexicality and the radical complexity not only affect writing preparation but also modulate the execution of writing movement, which provides support for a cascaded pattern of central and peripheral processing in written word production.

As expected, we found a radical boundary effect during writing movement; the lowest writing velocity was at the first stroke of the second radical and the fastest writing velocity was at the last stroke of the first radical. This pattern indicates that there was a significant increase in processing load at the first stroke of the second radical due to the planning of the second radical within a character. Correspondingly, there was a significant decrease in processing load at the last stroke of the first radical due to the absence of the planning of the second radical. Therefore, compared with the pattern of radical boundary effect observed in experiment 1, as the stroke number increases in the first radical, the preparation of the second radical is delayed.

### General discussion

This study investigated how the lexicality and the radical complexity affect central and peripheral processing in writing. We found that regardless of the lexicality, the participants were slower to initiate writing in the many-strokes condition than in the few-strokes condition. Interestingly, the lexicality and the radical complexity affected the pattern of non-stroke shift velocity and writing velocity during the writing execution. The experiments consistently found that the participants slow down their writing at the radical boundary strokes (S2 or S3), which suggested that the lexicality and the radical complexity affected both central processing and peripheral processing. This result indicates a cascaded pattern of written word production.

### The factors that influence central processing

In experiment 2, we found an effect of radical complexity but no effect of lexicality on writing latencies. The writing latencies were equivalent in the few- and many-strokes conditions in experiment 1 due to similar number of strokes in both conditions, indicating that the participants might programme the first radical before the writing execution. By contrast, the number of strokes had significant differences in the first and the second radicals between the few- and the many-strokes conditions in experiment 2. In other words, there was a larger difference between the few- and the many-strokes characters in experiment 2 than in experiment 1. Therefore, the effect of radical complexity was probably from the significant difference between the strokes number of the first radicals or of the whole characters, in the few- and the many-strokes characters. The correlation analysis further suggested that the participants might have prepared for the second radical before the initiation of writing execution.

The absence of the lexicality effect for the writing latency indicates that the lexicality does not affect the central processing. One may argue that the participants treat the real and non-characters similarly, which might occur because the characters used in experiment 2 were very rare. However, there were an effect of lexicality and an effect of the radical complexity on the non-stroke shift velocity during the interval from the offset of the last writing repetition to the onset of the next writing repetition. The radicals' positions were legal in the real characters but illegal in the non-characters. We thus suggest that the participants probably processed the radical positions information during the execution of the first repetition. This finding was confirmed by our findings in peripheral processing, which indicated a lexicality effect at S1 and S2 during the writing execution period (see below). After the first repetition, the lexicality effect occurred at the intervals as the participants prepared for the second and the third repetition.

### The factors that influence peripheral processing

The effect of the radical complexity on the writing velocity indicated a radical boundary effect where the writing velocity was slower at the radical boundary strokes (S2 and S3) compared to the non-radical boundary strokes (S1 and S4). We found a key difference between the two experiments in that the slowest velocity arose at S2 in experiment 1, whereas the slowest velocity was observed at S3 in experiment 2. Note that the characters used in experiment 1 were high frequency characters, while the characters used in experiment 2 were very rare. During the writing execution, the participants began to process the second radical while writing the first radical in the high frequency characters; in contrast, the participants started to process the second radical while writing the second radical in the rare characters. Our findings are consistent with the view that radicals are a processing unit in Chinese (i.e., Han et al., 2007). Therefore, we suggest that the execution of writing is dynamic and depends on the lexical properties and that the influence of the lexical properties (i.e., frequencies) cascades into peripheral processing in handwriting production (see also Delattre et al., 2006; Kandel et al., 2013).

The lexicality effect on the writing velocity was dependent on the stroke position; the writing velocity was slower in the characters than in the non-characters at S1 and S2 in the first radical, and equivalent at S3 and S4 in the characters and the non-characters. This finding indicates that the lexicality effect arises during the writing movement of the first radical, and not the second radical. Therefore, lexical processing cascades into peripheral processing but only at the beginning of the character. Roux et al. (2013) found that the lexicality effect on letter duration was largely dependent on the letter position and that the duration was longer for words at earlier letter position than pseudo-words. In other words, lexical processing was still active while the participants were writing the first and second letters, and the cascade ended at the third letter. The lexicality effect we observed in the present study is consistent with the findings by Roux et al. (2013).

For the stroke velocity in experiment 2, the velocity of S1 was as slow as the S3 in real characters while the former was faster than the latter in non-characters. The S1 belongs to the non-radical boundary strokes in the first radical while the S3 belongs to the radical boundary strokes in the second radical. It is hard to compare the difference between S1 and S2 when they varied in the lexicality and the stroke position. We speculate that when writing S1, the participants process some lexical properties (i.e., orthographic information) implicitly, and thus slow down their writing velocity in real characters, by contrast, the velocity of S1 in non-characters was fast. The analysis also indicates that the lexicality effect arises during the writing movement of the first radicals, and not the second radicals (see page 17 for details). After that, the processing load was increased because of processing the second radicals, the writing velocity of S3 was slow in real characters as well. By contrast, the writing velocity of S3 was slow down compared to when writing S1 in non-characters. Therefore, due to similar processing load (but on different properties) when writing S1 and S3 in real characters, we did not observed significant difference between them on writing velocity, by contrast, due to increased processing load when writing S3 in non-characters, we observed a significant difference between them on writing velocity. Although these differences between real and non-characters, they presented similar patterns in a whole: the lowest writing velocity arose at S3.

One limitation of the present study was the participants were asked to write characters stroke by stroke. The child learns to write Chinese characters in this way while adults usually write characters in a connective way. Due to this difference, adults may use a larger unit such as logographemes or radicals in central and peripheral processes in written production. We would expect more salient radical boundary effect in adults than in children. It is necessary to use a more natural writing way to examine processes of written production in further study.

In conclusion, our data suggests that lexicality and radical complexity processing operate during the execution of handwriting movements, which supports the idea that central processing cascades over peripheral processing during handwriting (Delattre et al., 2006; Álvarez et al., 2009; Roux et al., 2013). In addition, lexical processing cascades into peripheral processing but is limited to the beginning of Chinese characters. In contrast, radical complexity influenced the execution of handwriting movement throughout the entire character, and by combining the results of experiments 1 and 2, we see that the pattern of the effect interacts with the character frequency. Our findings reflect a cascaded pattern of written production in Chinese, which is similar to observations in other studies of different languages (e.g., French). We suggest that this cascaded pattern between central and peripheral processing is universal across different writing systems.

## Author contributions

QZ and CF proposed the ideas and designed the experiments. CF performed the experiments and analyzed the data. QZ wrote the manuscript.

### Conflict of interest statement

The authors declare that the research was conducted in the absence of any commercial or financial relationships that could be construed as a potential conflict of interest.
