# Supplementary material for: The Interaction between Central and Peripheral Processing in Chinese Handwritten Production: Evidence from the Effect of Lexicality and Radical Complexity
Source: Front Psychol. 2017 Mar 13;8:334. doi: 10.3389/fpsyg.2017.00334 (PMC5346556; doi:10.3389/fpsyg.2017.00334)
Supplement: Supplementary file 1 [file DataSheet1.docx]

**Appendix**

Stimuli used in Experiment 1

| with few-strokes | with many-strokes |
| --- | --- |
| 吠(fei4, bark) | 怵(chu4, afraid) |
| 杠(gang4, bar) | 呷(ga1, small drink) |
| 扛(kang4, carry) | 拣(jian3, pick up) |
| 圮(pi3, ruined) | 咀(ju3, chew) |
| 吻(wen3, kiss) | 棵(ke1, classifier) |
| 忻(xin1, inspire) | 悚(song3, thriller) |
| 妍(yan2, beautiful) | 咱(zan2, us) |
| 忖(cun3, think) | 怍(zuo4, shamed) |
| 叮(ding1, bite) | 拌(ban4, stir) |
| 抖(dou3, shake) | 埂(geng3, low bank) |
| 扶(fu2, support) | 哩(li3, miles) |
| 芊(qian1, luxuriant) | 咙(long3, throat) |
| 枉(wang3, vain) | 抹(mo3, wipe) |
| 忤(wu3, incompliance) | 坪(ping2, area) |
| 杖(zhang4, stick) | 哑(ya3,dumb) |

Stimuli used in Experiment 2

| Real Characters | | Pesudo-characters | |
| --- | --- | --- | --- |
| With  few-strokes | with  few-strokes | with few-strokes | With many-strokes |
| 饤(ding4,food for display) | 蛱(jia2,butterfly) | 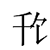 | 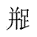 |
| 仏(fo2,budda) | 衉(ke4,spit) | 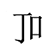 | 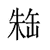 |
| 犴(han1,moose) | 豤(ken3,bite) | 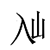 | 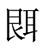 |
| 屸(hong2,mountain) | 聭(kui4,shameful) | 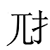 | 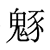 |
| 纩(kuang4,silf floss) | 躴(lang2,slender) | 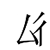 | 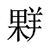 |
| 仂(le4,remainder) | 艃(li2,boat) | 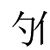 | 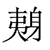 |
| 邙(mang2,mountain name) | 躶(luo3,naked) | 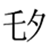 | 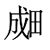 |
| 夘(mao3,mortise) | 缾(ping2,bottle） | 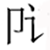 | 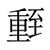 |
| 吀(mie1,bleating) | 酺（pu2,drink) | 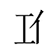 | 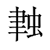 |
| 扖(ru4,inlay） | 粬(qu1,starter) | 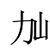 | 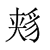 |
| 忕（shi4,be used to) | 貄(si4,beast) | 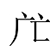 | 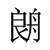 |
| 讬(tuo1,support) | 觫(su4,afraid) | 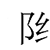 | 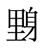 |
| 屼(wu4,towering) | 畽(tuan3,village) | 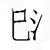 | 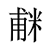 |
| 圯(yi2,bridge) | 臹(xiu1,practice) | 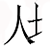 | 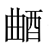 |
| 汄(ze4,flow) | 羠(yi2,mild sheep) | 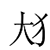 | 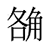 |
| 彴(zhuo2,single-plank bridge) | 跦（zhu1,jump) | 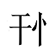 | 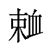 |
